# Exposure to silicone breast implant-infused media is detrimental to *Caenorhabditis elegans*

**DOI:** 10.17912/micropub.biology.000732

**Published:** 2023-02-08

**Authors:** Henry, B.P.M. Dijkman, Inca Slaats, Samantha Hughes

**Affiliations:** 1 HAN University of Applied Sciences, Nijmegen, the Netherlands; 2 Amsterdam Institute for Life and Environment, Environmental Health and Toxicology, Vrije Universiteit Amsterdam, Amsterdam, the Netherlands

## Abstract

Women are raising concerns about breast implant illness (BII), a collective term for a range of symptoms attributed to gel bleed. To study this, *Caenorhabditis elegans* was exposed to increasing duration of gel bleed from silicone breast implants (SBI) and the impact on health parameters observed. SBI exposure results in a slight reduction in total brood size with the progeny having impaired mobility. Nematodes displayed stress characteristics and silicones were detected inside the animals, suggesting silicone uptake after exposure to SBI. Our data highlights the need for more investigations into the mechanisms and pathways impacted by SBI.

**Figure 1. Effects of silicone breast implant infused media on adverse outcomes in C. elegans:**
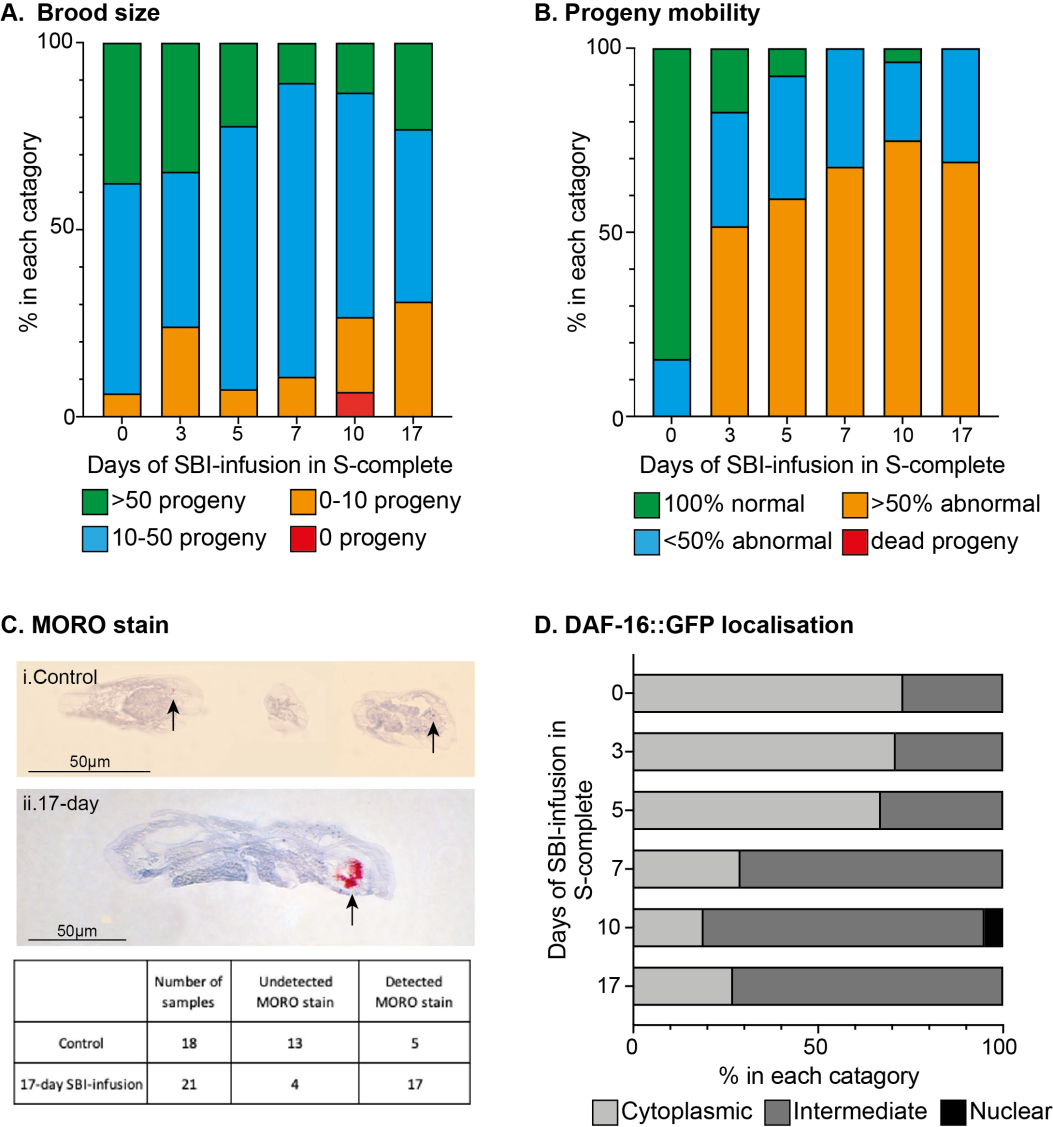
(A) Brood size was estimated after 48-hour exposure to SBI-infused media. The number of progeny was classified into bins, where there was expected to be more than 50 viable offspring in control conditions (bin 3, green bars). If 10-50 progeny were produced, this was shown with blue bars (bin 2). Orange bars (bin 1) indicate up to 10 viable offspring and red bars are indicative of no viable progeny (bin 0). No acute toxicity of the parents was observed in any condition. For each condition, n > 26 from 2 independent biological replicates and the percentage of worms in each bin is shown. A chi ^2^ test for homogeneity resulted in a *p* =0.01. (B) The health of the offspring was assessed by observing their mobility. A healthy worm displays a rapid head-to-tail body bend and, this is observed in 100% of the offspring (bin 3, green bars) or in less than 50% of the offspring (bin 2, blue bars). Abnormal mobility was noted when the worms had an uncoordinated thrashing motion which is indicative of neurotoxicity, shown by orange bars (bin 1) and dead nematodes are indicated with red bars (bin 0, there were none in this category). For each condition, n > 26 from 2 independent biological replicates and the percentage of worms in each bin is shown. Statistical analysis (chi ^2^ test) gives *p* <0.0001. (C) Modified Oil Red O (MORO) staining of *C. elegans* indicates that silicones are located in cryosections of 48-hour exposed animals. (i) Three sections of *C. elegans* exposed to control conditions. Some small areas of positive MORO stain are indicated by arrows. (ii) Animals exposed to 17-day SBI-infused S-complete show a clear and distinctive MORO stain, that is positive for silicone, highlighted with an arrow. Scale bar is 50µm. A table to show the number of samples which had MORO stain present (detected) or not (undetected). In the control worms, the detected MORO stain is at such low levels this is considered background. (D) Nematodes are placed on silicone breast implant infused NGM as L4s and incubated at 20 ^o^ C for 48 hours. The cytoplasmic expression of DAF-16::GFP (light grey bars) is a result of the absence of stress. As the conditions become more stressful, the expression of the reporter becomes more nuclear (black bars). Intermediate localisation (dark grey bars) indicates some animals are stressed as their DAF-16::GFP can be found in both the nucleus and cytoplasm in the same worm. For each condition, n > 30 in 1 biological replicate. The percentage of worms in each category is shown with *p* <0.001 indicated by a chi ^2^ test for homogeneity.

## Description

It has been shown that silicone leakage, also known as gel bleed, occurs in 99% of women with silicone breast implants (SBI) and 87% of these women had silicone particles beyond the breast tissue (Dijkman et al. 2021). Gel bleed may lead to Breast Implant Illness (BII), also known as silicone toxicity (Brawer 2019; Wee et al. 2020) as well as Breast Implant-Associated Anaplastic Large Cell Lymphoma (BIA-ALCL) and squamous epithelial carcinoma/lymphoma (Deva et al. 2020; Fleury and D’Alessandro 2021). To date the literature concerning BII is descriptive and observational, however many women are actively removing their breasts to reduce implant-associated illnesses (Brawer 2017; Habib et al. 2022; Spit et al. 2022; Wee et al. 2020). Despite the increased awareness of silicone toxicity and BII, there are very few scientific papers that address the toxic effects as a consequence of gel bleed in people with SBIs. Due to the challenges associated with exploring the effect of gel bleed from implants, it would be ideal to explore the effects in a whole-organism model. Therefore, we mimicked gel bleed and explored the toxic effect in a relevant model system, the nematode *Caenorhabditis elegans* .

Silicone toxicity has been investigated in *C. elegans* in relation to silicone nanoparticles (Eom and Choi 2019; Lee et al. 2020; Zhang et al. 2020), although such studies use a different type of silicone compared to those found in SBIs. We wished to utilise the fact that *C. elegans* can be grown in liquid media to explore how gel bleed from SBIs impact development and reproduction. To replicate leakage from the breast implant, the content of the implant was added to S-complete (Heshof et al. 2019) for increasing amounts of time.

Here, *C. elegans* were exposed to S-complete (Heshof et al. 2019) which had been infused with SBI for increasing amounts of time, mimicking the effect of SBI gel bleed, rupture and leakage in a human patient, allowing the assessment of acute toxicity and changes in reproduction and development. The SBI-infused S-complete was used as the liquid media in which nematodes were placed, and after 48 hours exposure the brood size and development of the progeny observed. For a control, worms were exposed to S-complete alone. The nematodes exposed to the SBI-infused S-complete showed no acute toxicity but did have a small but significant reduction ( *p* =0.01) in total brood size when exposed to media infused with SBI (Figure 1A). When observing the progeny for an impaired head-to-tail thrash, as a proxy for health of the progeny, we found that in all SBI-infused media conditions, more than 50% of the progeny displayed a significant defect in mobility compared to control conditions *p* <0.001 (Figure 1B).

To assess if the worms took up the silicone from the solution, a modified Oil Red O (MORO) stain was applied to cryosections of nematodes exposed to SBI-infused media (Dijkman et al. 2021; Kappel et al. 2016; Mustafa et al. 2022). Nematodes at the L4 stage were exposed to the SBI-infused media from two conditions (control and 17-day SBI-infused media). After 48 hours, worms were washed off the plates, pelleted and cryosections prepared (Dijkman et al. 2021; Jänes et al. 2018). MORO stain was applied to assess for the presence of silicones within the worm tissue. There was a clear increase in the positive staining for silicone particles in nematodes that had been subjected to SBI-infused media (Figure 1C). It is important to note that 28% of the negative control samples did have small MORO positive stained areas, which was at such low levels they are thought to be background. As expected, the number of samples positive for MORO stain was increased in nematodes that were exposed to the 17-day SBI-infused media (81% positive samples). The MORO stain in these samples was very clear and distinctive, covering a large area. Together, this suggests that silicone is taken up by the nematodes following exposure to SBI. Due to the preparations of nematode material for cyrosectioning, it was not possible to identify the specific tissues in which the silicones were located at this time. However, it is significant that this data demonstrates that the worms do take up the silicone, with more silicone staining observed in the worms exposed to a longer period of SBI-infused media, similar to that in humans (Dijkman et al. 2021).

The data from MORO staining suggests that silicones have been taken up by the nematodes, but this raises some questions as to which molecular pathways are being altered by exposure to the silicone, and other compounds from the SBI. To explore this, we used a fluorescent reporter nematode strain to observe stress. DAF-16 is the nematode homolog of FoxO, a conserved transcription factor (Martins et al. 2016) that is heavily implicated in the regulation of lifespan and stress resistance (Henderson and Johnson 2001; Sen et al. 2020; Senchuk et al. 2018). By using a transgenic strain, TJ356, that expresses the fusion protein DAF-16::GFP, fluorescence could be detected in the cytoplasm or nucleus, with more nuclear localisation suggesting activation of stress response pathways (Büchter et al. 2013). As expected, the majority of the worms in control conditions (i.e., not exposed to the SBI-infused S-complete) had cytoplasmic localisation of DAF-16::GFP. After 48 hours exposure to the 3-day SBI-infused NGM, there was similar levels of stress in the worms, with worms exposed to 17-day SBI-infused media, the majority of worms (70%) have more nuclear expression, suggesting that in these conditions the worms are more stressed. (Figure 1D).

The U.S. Food and Drug Administration (FDA) has recently taken action to warn about the increased risk of complications from breast implants, highlighting the urgency to have a clear understanding of how SBIs impact health. As the safety of SBIs is questioned, women should be well informed about the potential harms before these implants are placed into the body. Monitoring must be intensified across the lifespan of the implant and throughout the life of the person with SBIs.

Using the well-described model system *Caenorhabditis elegans* and a method to represent gel bleed, the worms have a small reduction in fertility, with the progeny displaying impaired mobility as a result of exposure to the SBI-infused media. Additionally, there are indications that the worms have a stress response to the SBI-infused media in a time-dependent manner. While further research is required to fully investigate the pathways and mechanisms resulting in the nematode’s stress response to SBIs, we are the first to note how SBI induce toxicity in a whole-organism system.

## Methods

**Strains and maintenance** *C. elegans* strains were maintained at 20 ^o^ C on NGM plates with *E. coli* strain OP50 as a food source.

**Replication of silicone breast implant leakage** To replicate gel blead, SBI-infused S-complete was prepared. S-complete was prepared (Heshof et al. 2019; Solis and Petrascheck 2011) and 50ml added to a 50ml Falcon Tube. To the solution, 0.5g of silicone breast implant, provided by Henry Dijkman, was added. The tubes were left gently rotating at room temperature (19-21 ^o^ C) for 3, 5, 7, 10 or 17 days before they were used in assays.

**Developmental and reproductive toxicity** The effect of the SBI on the development and reproduction of the nematodes was assessed using a liquid assay (Hughes et al. 2022). SBI-infused S-complete was used to resuspend a pellet of concentrated *E. coli* . Then, 50µl of this solution is added to a well of a 96-well plate followed by a single L4-stage worm in 10µl M9 buffer. Each row of the 96-well plate has a different “condition” with up to 12 replicates per condition. Each plate contained a negative control, which is the S-complete which was not exposed to the SBI. The plates were incubated at 20 ^o^ C with gentle shaking at 150 rpm for 48 hours. After 48 hours, each well (i.e., a single nematode or statistical unit) was assessed for acute toxicity of the parent, as well as the approximate brood size produced. To assess reproduction, an estimate of the brood size was classified into bins. The bins were as follows: “3” where there were more than 50 viable progeny, essentially wild type; “2” with between 10-50 progeny; “1” indicated up to 10 progeny; “0” had no progeny. The impact of the SBI-infused media on the development of the progeny in each well was observed and classified into bins. Bin “3” indicated the situation where all progeny develop normally; “2” was a bin where less than 50% of the offspring display abnormal behaviour; “1” indicates that more than 50% of the progeny have mobility defects; “0” is where the progeny are dead as indicated by nematodes having a rod-like appearance. Data was collected from two independent biological replicates, each with at least 12 individuals at each “concentration” of SBI-infusion. The data was combined and plotted in GraphPad Prism as percentage stacked bar charts. A chi ^2^ test for homogeneity was undertaken. For brood size, bins 0 and 1 were merged, and for progeny mobility bins 0, 1 and 2 were merged to meet the validity conditions for the test.

**DAF-16::GFP localisation** For the DAF-16::GFP localisation assay, worms that express the DAF-16::GFP reporter (strain, TJ356) were age synchronised and were added to SBI-supplemented NGM as L4s. After 48 hours incubation at 20 ^o^ C, the sub-cellular localisation of GFP was assessed using a Leica M165FC microscope at x20 magnification. The localisation was classified as cytosolic (no stress), intermediate or nuclear (stressed) and the data presented as a percentage of the population in each category, from *n* > 30 in one biological replicate, with a chi ^2^ test for homogeneity performed.

**Observing silicone distribution using MORO staining** Age synchronised L4 worms are added to NGM plates supplemented with SBI-infused S-complete. Infusion of the S-complete was for 5 and 17 days. Worms were incubated at 20 ^o^ C for 48 hours and washed off the plates using M9 buffer before being pelleted by centrifugation at 2500 rpm for 90 seconds. The pellet was washed 3 times before being dropped from a glass pipette into liquid nitrogen to form “popcorn” and stored at -80 ^o^ C until needed (Jänes et al. 2018). To stain the samples, the pellets were blocked in Tissue-Tek and cut by cryosectioning into 2µm and 10µm slices, air-dried and fixed in 4% formalin for 10 minutes followed by 5 minutes in 1x PBS, according to published methods (Dijkman et al. 2021; Kappel et al. 2016). A modified Oil Red O protocol was used to detect silicones in *C. elegans* tissues (Dijkman et al. 2021; Mustafa et al. 2022). Oil Red O (Merck 1.05230.0025) was added to 1,2-propanediol and heated at 95 ^o^ C with continuous mixing for 1 hour. Once cool, the solution is filtered through 185mm Whatman filter paper. The cryosections are flushed with water for 10 minutes and then placed in 100% 1,2-propanediol for 5 minutes. Slides were transferred to the MORO stain and incubated for 3 days at 4 ^o^ C. After incubation, cryosections are washed with 85% 1,2-propanediol for 2 minutes and flushed with water. Lastly, tissues are counter stained with Haemotoxylin Mayer (HE) for 10 minutes and then washed with water for a further 5 minutes. The sections are briefly places in 1% acetic acid (to prevent background staining) and washed with water for 4 minutes. Cover slips are added when the sections are still wet and mounted in Fluoromount-G (Thermo Fisher) then observed using a Leica DCF7000T at x40 magnification and the Zeiss Imager.M2.

## Reagents

**Table:** 

Strain name	Genotype	Available from
N2	*Caenorhabditis elegans*	CGC
TJ356	*zIs356* IV ( *pdaf-16::daf-16::gfp* + *rol-6* )	CGC
